# Glutathione Peroxidase 5 Is Expressed by the Entire Pig Male Genital Tract and Once in the Seminal Plasma Contributes to Sperm Survival and *In Vivo* Fertility

**DOI:** 10.1371/journal.pone.0162958

**Published:** 2016-09-14

**Authors:** Isabel Barranco, Asta Tvarijonaviciute, Cristina Perez-Patiño, Alejandro Vicente-Carrillo, Inmaculada Parrilla, Jose J. Ceron, Emilio A. Martinez, Heriberto Rodriguez-Martinez, Jordi Roca

**Affiliations:** 1 Department of Medicine and Animal Surgery, Faculty of Veterinary Science, University of Murcia, Murcia, Spain; 2 Department of Clinical and Experimental Medicine (IKE), University of Linköping, Linköping, Sweden; Universite Blaise Pascal, FRANCE

## Abstract

Glutathione peroxidase-5 (GPX5) is an H_2_O_2_-scavenging enzyme identified in boar seminal plasma (SP). This study attempted to clarify its origin and role on sperm survival and fertility after artificial insemination (AI). GPX5 was expressed (Western blot and immunocytochemistry using a rabbit primary polyclonal antibody) in testes, epididymis and accessory sex glands (6 boars). SP-GPX5 concentration differed among boars (11 boars, P < 0.001), among ejaculates within boar (44 ejaculates, P < 0.001) and among portions within ejaculate (15 ejaculates). The first 10 mL of the sperm rich fraction (SRF, sperm-peak portion) had a significantly lower concentration (8.87 ± 0.78 ng/mL) than the rest of the SRF and the post-SRF (11.66 ± 0.79 and 12.37 ± 0.79 ng/mL, respectively, P < 0.005). Sperm motility of liquid-stored semen AI-doses (n = 44, at 15–17°C during 72h) declined faster in AI-doses with low concentrations of SP-GPX5 compared to those with high-levels. Boars (n = 11) with high SP-GPX5 showed higher farrowing rates and litter sizes than those with low SP-GPX5 (a total of 5,275 inseminated sows). In sum, GPX5 is widely expressed in the boar genital tract and its variable presence in SP shows a positive relationship with sperm quality and fertility outcomes of liquid-stored semen AI-doses.

## Introduction

Boar spermatozoa are especially sensitive to oxidative stress (OS) induced by reactive oxygen species (ROS) due to the large proportion of polyunsaturated fatty acids (PUFA) in the plasma membrane and the low intracytoplasmic concentrations of ROS-scavengers [1]. The OS leads to lipid peroxidation (LPO) resulting in disruption of sperm functionality and impairing fertilizing ability [2]. Seminal Plasma (SP) protects sperm against the negative impact of ROS as it contains ROS-scavengers, including antioxidant enzymes [3, 4]. Many of these ROS-scavengers, but not antioxidant enzymes, can be jointly measured using Total Antioxidant Capacity (TAC) assays [5] and a positive relationship between SP-TAC and fertility outcomes of boars included in artificial insemination (AI) programs has recently been proven [6].

Hydrogen peroxide (H_2_O_2_) is considered as the major damaging ROS for boar sperm [7,8]. When in excess, H_2_O_2_ is partially reduced to hydroxyl radical (·OH), attacking sperm membranes, particularly PUFAs, leading to sperm dysfunction and ultimately to sperm death [9]. Glutathione Peroxidases (GPXs) and Catalase (CAT) are the enzymes responsible for neutralizing H_2_O_2_ by reducing it to water. In contrast to the powerful H_2_O_2_ recycling CAT enzyme, the GPX-family acts more as intra- and extra-cellular H_2_O_2_ regulator alongside acting as repairing enzymes by recycling organic peroxidized molecules, particularly those in membrane PUFA [10]. The GPX-family includes up to a total of eight phylogenetically related enzymes; GPX1-8. Among these the GPX5 is particularly attractive for male reproduction as it represents more than 95% of the total GPXs present in the epididymal fluid [11]. Looking at boar ejaculates, controversy exists about the relationship between SP-GPX5 and sperm fertilizing ability. Novak et al. [12] reported a positive relationship between SP-GPX5 and farrowing rates, whereas more recently Vilagran et al. [13] found a negative relationship between SP-GPX5 and sperm membrane integrity and motility parameters in fresh ejaculated sperm. These findings should be interpreted with caution because, as indicated by the authors themselves, the results come from experiments either preliminary or not particularly focused on GPX5, thus calling for further confirmatory research. Consequently, the present study has as primary purpose to elucidate the relationship between SP-GPX5 and boar sperm quality, including fertility in liquid-stored AI semen doses of a large number of boars used for AI. Alongside, it intends to determine if GPX5 is synthesized in other parts of the boar reproductive tract besides the epididymis and; finally, to evaluate the variability in SP-GPX5 among boars and even within the ejaculates of the same boar, including differences among ejaculate portions. Semi-automatic collection systems are now successfully replacing the classical manual semen collection procedures (gloved-hand method), arguing hygienic and labor-cost reasons [14]. Use of these novel systems necessarily implies collection of the entire ejaculate in a single vial, thus deviating from the *in vivo* mating situation where ejaculate fractions enter the female in sequence. The procedure also increases the proportion of SP (mainly derived from the post-sperm rich ejaculate fraction [post-SRF]) for semen to be processed for AI, in contrast to previous collections of the SRF-only [14], which was hereby used for semen processing and elaboration of AI-doses. Consequently, the ejaculate portions hereby explored were manually collected using the gloved-hand method; (i) the first 10 mL of SRF where the dominant SP-fluid derives from the cauda epididymis, as well as (ii) the rest of SRF and (iii) the post-SRF, where the SP is mainly derived from accessory sexual glands.

## Material and Methods

### Reagents and media

All chemicals used in the experiments were of analytical grade. Unless stated otherwise, all media components were purchased from Sigma-Aldrich (St. Louis, MO, USA), and the media were prepared under sterile conditions in a laminar flow hood (MicroH, Telstar, Terrasa, Spain). Fluorochrome molecules were purchased from Molecular Probes (Europe BV, Leiden, The Netherlands).

The basic media used to dilute reagents and fluorochromes for flow cytometry was EDTA-free phosphate-buffered saline (PBS: NaCl 139 mM, KCl 2.7 mM, KH_2_PO_4_ 1.5 mM, Na_2_HPO_4_·7H_2_O 8.1 mM; with 0.058 g/L penicillin G and 0.05g/L streptomycin sulphate; pH 7.1 ± 0.03; 286 ± 7 mOsmol/kg).

### Animals and ejaculates

All procedures that involved animals were performed according to international guidelines (Directive 2010-63-EU) and approved by the Bioethics Committee of Murcia University (research code: 639/2012).

Ejaculates (Exps 1, 3–4) and organ samples (see Exp 2) were obtained from healthy, sexually mature boars of different breeds or crossbreds undergoing regular semen collection for commercial AI. Boars were housed in 4 Spanish insemination centers belonging to AIM Ibérica (Topigs Norsvin España) and located in León, Lerida, Murcia and Soria provinces. All boars were subjected to the same housing conditions, specifically individual pens in environmentally controlled (15–25°C) buildings with windows so that they were exposed to natural daylight and supplementary light for a total of 16 h of light per day. They were provided with *ad libitum* access to water and were fed commercial feedstuff, according to nutritional requirements for adult boars. All ejaculates used fulfilled the standards of quantity and sperm quality thresholds for the preparation of semen AI-doses (more than 200 x 10^6^ spermatozoa/mL, 70% motile spermatozoa, and 75% of morphologically normal cells). In addition, boars included in the study were periodically (every 4 months) subjected to analysis of sperm nuclear DNA fragmentation, using a commercial variant of the Sperm Chromatin Dispersion test specifically designed for boar spermatozoa (Sperm-Sus-Halomax^®^; Halotech DNA SL, Madrid, Spain), and fragmentation rates were consistently below 3% in all boars.

### Seminal plasma processing and storage

Seminal plasma samples were obtained through double centrifugation (1,500xg for 10 minutes) (Rotofix 32A, Hettich Zentrifugen, United Kingdom) immediately at ejaculation. After the second centrifugation, the supernatant was harvested and examined by microscopy to ensure it was sperm-free to be thereafter stored in cryotubes and sent in insulated containers with ice dry to the Andrology Laboratory at the Veterinary Teaching Hospital of the University of Murcia. At the laboratory, the SP samples were stored at -80°C (Ultra Low Freezer, Haier, Canada), until analyzed. For analyses, the SP samples were thawed at room temperature.

### Measurement of SP-GPX5 concentration

The SP-GPX5 concentration was measured using a commercially available sandwich enzyme-linked immunosorbent assay following manufacturer’s instructions (Mybiosource, San Diego, California, USA). This assay employs a pre-coated microplate with monoclonal antibodies specific for pig GPX5. Briefly, standards for the standard curve (ranging from 0.625–40 ng/mL) and SP-samples were added in duplicate to wells (100 μL) to bind GPX5 to the immobilized antibodies. In blank wells, sample diluent (100 μL) was added. The plates were incubated for 120 min at 37°C in darkness. After that, the contents of each well was aspirated and washed 3 times with wash buffer (250 μL) using an automated plate washer (ELx50/8RDS, BioTek Instruments, Winooski, VT, USA). The plate was blotted against clean paper towels to remove any remaining wash buffer, and 100 μL of biotin-conjugated antibody specific for GPX5 (diluted 1:100 in Biotin-Conjugate diluent) was added to the wells and incubated for 60 min at 37°C in darkness. The plate was again washed as described above, prior to the addition of 100 μL of Streptavidin conjugated Horseradish Peroxidase (diluted 1:100 in Streptavidin-Horseradish Peroxidase diluent) and then incubated at 37°C in darkness for 1h. Following another wash (5 times), Substrate solution (100 μL) was added to the wells and incubated for 20 min at 37°C in darkness. A color change was shown in proportion to the amount of bound GPX5. Subsequently, Stop solution (50 μL) was added to each well, and the optical density of the amount of substrate converted to product was detected at 450 nm using a micro-plate reader (PowerWave XS; Bio-Tek Instruments) within 5 min after the stop solution addition, with a correction wavelength set at 590 nm. Values were expressed as ng/mL.

### Assessment of sperm quality

The spermatozoa were assessed according to quality (total and progressive motility and viability) parameters. Sperm motility was evaluated using a computer-assisted sperm analyzer (CASA) and sperm viability was assessed by flow cytometry using a BD FACS Canto II flow cytometer (Becton Dickinson & Company, Franklin Lakes, NJ, USA). Hoechst 33342 (H-42) fluorescence (DNA content) was used for identifying sperm events. Acquisition was stopped after 10,000 H-42 positive events.

Sperm motility was objectively evaluated using an ISASV1^®^ CASA (Proiser R+D, Paterna, Spain). For each evaluation, a 5 μL of extended semen (20–30 x 10^6^ spermatozoa/mL in Beltsville Thawing Solution) was placed in a 10 μL-Makler counting chamber (Sefi Medical Instruments, Haifa, Israel) that had been pre-warmed to 38°C, and six to nine fields, with a minimum of 400 spermatozoa per sample, were analysed. The sperm motility variables recorded were the overall percentage of motile spermatozoa (average path velocity ≥20 μm/sec) and the proportion of motile spermatozoa showing rapid and progressive movement (straight line velocity ≥40 μm/sec).

For sperm viability assessment, 100 μL of semen (30 x 10^6^ spermatozoa/mL in PBS) were mixed with 3 μL H-42 (0.05 mg/mL in PBS), 2 μL propidium iodide (PI, 0.5 mg/mL in PBS), and 2 μL fluorescein-conjugated peanut agglutinin (PNA-FITC, 100 μg/mL in PBS) and then incubated at 38°C in the dark for 10 min. Immediately before analysis, 400 μL of PBS were added to each sample. Viable spermatozoa were those exhibiting intact plasma and acrosome membranes (H-42 positive/PI negative and PNA-FITC negative) and were reported as percentages.

### Western blotting (WB)

Proteins were extracted in RIPA buffer (Sigma-Aldrich, Sweden) at 4°C for 40 min, by sonication (Skafte Medlab ultrasound bath; Bandelin Sonorex, Digitec, Berlin, Germany) at 35 kHz for 30 min in ice. After centrifugation at 13,000xg for 10 min, the supernatant was processed for protein quantification (DC Protein assay kit, Bio Rad, USA) and dilution to a final concentration of 25 μg protein/10 μL for WB using a rabbit primary polyclonal antibody (ab190733, Abcam, dilution 1:1000) against GPX5 (epididymal androgen-related protein), following the protocol of Vicente-Carrillo et al. [15].

### Immunohistochemical examination (IHC)

The sections of tissue samples were immunohistochemically stained using an avidin-biotin complex technique (Vector Laboratories, Burlingame, CA, USA). The sections were deparaffinized by washing three times with Histo-Clear (Histolab, Gothenburg, Sweden), progressively rehydrated from 100 to 50% ethanol, and placed in distilled water. All washing between incubations was performed with PBS-Tween (0.01%), with a final wash in distilled water. Antigen retrieval was performed in 10 mM Tris–1 mM EDTA (pH 9.5) and microwave exposure for 20 min. Sections were incubated overnight at 4°C with the same rabbit primary polyclonal antibody (ab190733, Abcam) as for WB, diluted in PBS containing 3% normal goat serum blocking solution (Dako, Stockholm, Sweden) and 1% Triton X-100. As negative control the primary antibody was omitted while sections of oral lamina propria (blood vessels) were used as positive controls. After washing, sections were incubated with a polyclonal goat anti-rabbit secondary antibody conjugated with biotin (Dako, Stockholm, Sweden), diluted 1:300 in PBS containing 3% normal goat serum blocking solution and 1% Triton X-100 for 30 min. Sections were washed, and endogenous peroxidase activity was blocked by incubating the sections with 3% H_2_O_2_ for 20 min. The immunostaining was developed using the VECTASTAIN Elite ABC kit and ImmPACT DAB Peroxidase (HRP) Substrate (Vector Labs, Burlingame, USA). The immunostained slides were mounted with Immuno-HistoMount prior visualization and photography, which were performed using an upright Olympus BX51 light microscope, equipped with phase contrast optics and a XC30 camera, and the CellSens Olympus imaging software (Olympus, Münster, Germany). The immunostaining at slide level was ranked by three independent observers to determine eventual changes in distribution or subjective intensity among the boars examined (n = 6). A simple ranking system (0: no staining, +-+++), was used to depict the various degrees of immune stating intensity and averaged between observers.

### Experimental design

#### Experiment 1: Inter-boar, intra-boar and intra-ejaculate variability in SP-GPX5 concentration

GPX5 concentration was quantified in SP samples from 44 entire ejaculates (4 ejaculates of 11 boars) to investigate the variability among boars (inter-boar variability) and within the same boar (intra-boar variability). In order to investigate intra-ejaculate variability, SP-GPX5 concentration was measured in three main ejaculate portions of each boar ejaculate: the first 10 mL of SRF (sperm-peak portion), the rest of SRF and the post-SRF, from 15 ejaculates (1 ejaculate per boar). Entire ejaculates were collected in one single vial using a semi-automatic collection method (Collectis®) while separate ejaculate portions (sperm-peak, rest of the SRF and the post-SRF) were collected using the regular gloved-hand method.

#### Experiment 2: Western blot and immunohistochemistry of GPX5 expression in boar testis, epididymis, spermatozoa and accessory glands

A total of six healthy, sexually mature boars of proven fertility (aged 18 to 36 months) of different breeds were used in this experiment. The boars were slaughtered (slaughterhouse La Mata de los Olmos, Teruel, Spain) for reasons of genetic replacement, without any relation to health or fertility issues. Shortly after slaughter, the scrotal contents and the internal genital tract were dissected out to collect tissue samples (1 cm x 1 cm and 1 mm thick) from medial testis, corpus and cauda epididymis, and mid-areas of the prostate, the seminal vesicle and the bulbourethral gland. Samples were immediately frozen by plunging in liquid nitrogen (Western blot [WB]) or immersion-fixed in 4% phosphate-buffered formalin, processed for paraffin embedding and sectioned (Inmunohistochemical analysis). Sections of 4 μm thickness were obtained and mounted on Superfrost Plus slides (Thermo Scientific, Gothenburg, Sweden). The samples were immunostained as described in the corresponding section. Frozen samples were stored at -80°C until processing.

#### Experiment 3: Relationship between SP-GPX5 concentration and quality of liquid-stored semen samples

Semen samples from 44 ejaculates (4 per boar) extended in commercial extender (Biosem+, Magapor, Zaragoza, Spain) at 3 x 10^9^ sperm/mL, alike conventional commercial AI-doses, were stored at 15–17°C for 72 h. Sperm quality was evaluated at 24 and 72 h of storage. A SP sample of each of the 44 ejaculates was used for measuring SP-GPX5 concentration.

#### Experiment 4: Relationship between SP-GPX5 concentration and fertility post-AI of liquid-stored semen samples

Weaned multiparous (1–7 farrows) Landrace and Large White sows housed in different farms of Spain were cervically inseminated (2–3 times per oestrus) using 24–72 h liquid-stored AI-semen doses (2,500 x 10^6^ sperm in 80 mL) prepared from entire ejaculates collected from 11 boars. The number of inseminated sows per boar ranged between 174 and 904. Fertility parameters were recorded over a 12-month period in terms of farrowing rate and litter size (total number of piglets born per litter) and SP-GPX5 concentration was measured in four ejaculates per boar from those used for AI purpose (one sample each 3 months).

### Statistical analysis

Data were statistically analysed using IBM SPSS Statistics 19.0 (IBM Spain, Madrid). The residual data for each statistical variable were evaluated using the Kolmogorov-Smirnov test to check the assumption of normality, and those not normally distributed were arcsine- (data in percents) or log- (count data) transformed. In Exp.1, a mixed ANOVA including the effects of boar and ejaculate within boar was performed to investigate inter- and intra-boar variability on SP-GPX5 concentration, and intra-boar reliability was assessed by intra-class correlation [ICC (3,1)] in a two-way mixed approach. Differences in SP-GPX5 concentration among ejaculate portions were assessed using a one-way ANOVA. In Exp.3, a hierarchical cluster analysis was carried out to identify naturally occurring groups within the SP-GPX5 data set, identifying two groups as high and low SP-GPX5 concentration. A one-way ANOVA was performed to verify the extent of the differences on SP-GPX5 concentration between the two groups. Then, a repeated-measures ANOVA was performed to evaluate the influence of SP-GPX5 group and storage time (24 and 72 h) on sperm quality parameters. In Exp.4, to analyze the relationship between SP-GPX5 and boar fertility parameters, the raw fertility dataset was corrected for parameters related to farm and sow by using the multivariate statistical model previously described by Broekhuijse et al. [16]. A hierarchical cluster analysis was also carried out to identify naturally occurring groups within the SP-GPX5 data set of 11 boars, identifying two groups of boars showing high and low SP-GPX5 concentrations. A one-way ANOVA was performed to investigate differences on fertility parameters between the two groups of boars. Nonparametric Receiver operating characteristic (ROC) curve was used to determine the value of the SP-GPX5 concentration to discriminate between boars showing high or low fertility outcomes. Areas under the curve (AUC) and cut-off values were selected by the program. The discrimination strength was measured by the AUC according to the following ranges: 0.90–1 = excellent, 0.80–0.90 = good, 0.70–0.80 = fair, 0.60–0.70 = poor, 0.50–0.60 = fail and 0.0–0.50 = no discrimination value. The Bonferroni test was used for post-hoc analyses where appropriate. A value of P < 0.05 was accepted as the minimal level of significance. Data are shown as means ± standard error of the mean (SEM).

## Results

### Experiment 1: Inter-boar, intra-boar and intra-ejaculate variability in SP-GPX5 concentration

**Fig 1:**
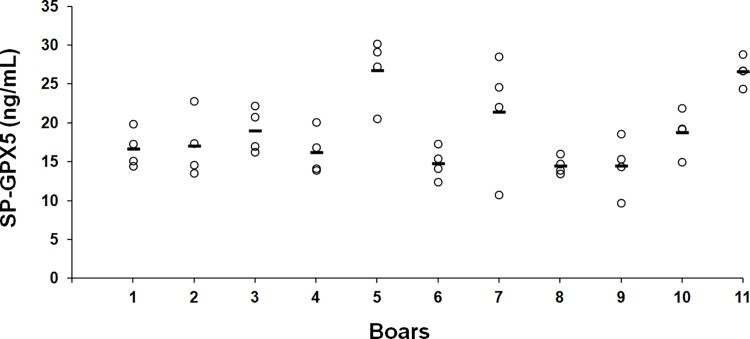
Variation among boars and among ejaculates within boar in the concentration of Glutathione Peroxidase 5 in seminal plasma (SP-GPX5). Scatterplot showing the concentration of SP-GPX5 of entire ejaculates collected from 11 boars (four ejaculates per boar). Circles show the SP-GPX5 concentration measured in each ejaculate and the lines show the mean for each boar.

The SP-GPX5 concentration showed significant (P < 0.001) inter-boar variation, with boar numbers 8 and 9 showing the lowest and boar numbers 5 and 11 the highest concentrations (Fig 1). SP-GPX5 concentration showed significant intra-boar variability (P < 0.001). However, the inter-boar variability (F-values = 28.04, df = 10) was considerably greater than the intra-boar variability (F-values = 4.73, df = 33). The ICC score was 0.85 (CI: 0.63–0.96; 95%), indicating good consistency in the measurements of SP-GPX5 concentrations among ejaculates within boar.

**Fig 2:**
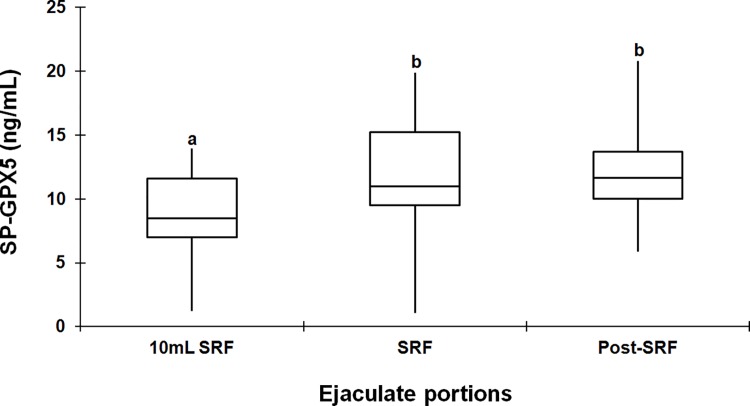
Variation in the concentration of Glutathione Peroxidase 5 (SP-GPX5) in seminal plasma among the three different portions of the boar ejaculate. Box-whisker plot showing variation in the concentration of SP-GPX5 in the three-ejaculate portions: the first 10 mL of spermatozoa-rich ejaculate fraction (SRF, sperm-peak portion), the rest of SRF and the post-SRF of 15 ejaculates (one per boar). Boxes enclose the 25th and 75th percentiles; the line is the median; and the whiskers extend to the 5th and 95th percentiles. (a,b) indicate significant differences (P < 0.005) among ejaculate portions.

SP-GPX5 concentrations varied significantly (P < 0.005) among ejaculate portions, the first 10 mL of SRF (sperm-peak) showing lower concentrations (8.87 ± 0.78 ng/mL, range: 7.13–10.60) than the rest of SRF (11.66 ± 0.79 ng/mL, range: 9.91–13.40) and the post-SRF (12.37 ± 0.79 ng/mL, range: 10.62–14.11). There were no differences between the concentrations measured in the rest of SRF and post-SRF (Fig 2).

### Experiment 2: Western blot and immunohistochemistry of GPX5 expression in boar testis, epididymis, spermatozoa and accessory glands

**Fig 3:**
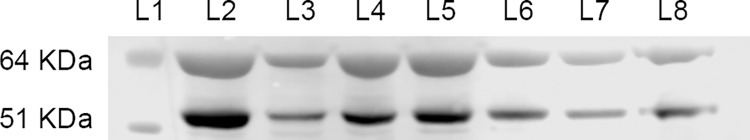
Western blot detection of GPX-5 in boar genital tract. L1: protein ladder; L2: blood vessel (positive control); L3: testis. L4: epididymis. L5: cauda epididymis. L6: seminal vesicle. L7: prostate. L8: bulbourethral gland.

**Fig 4:**
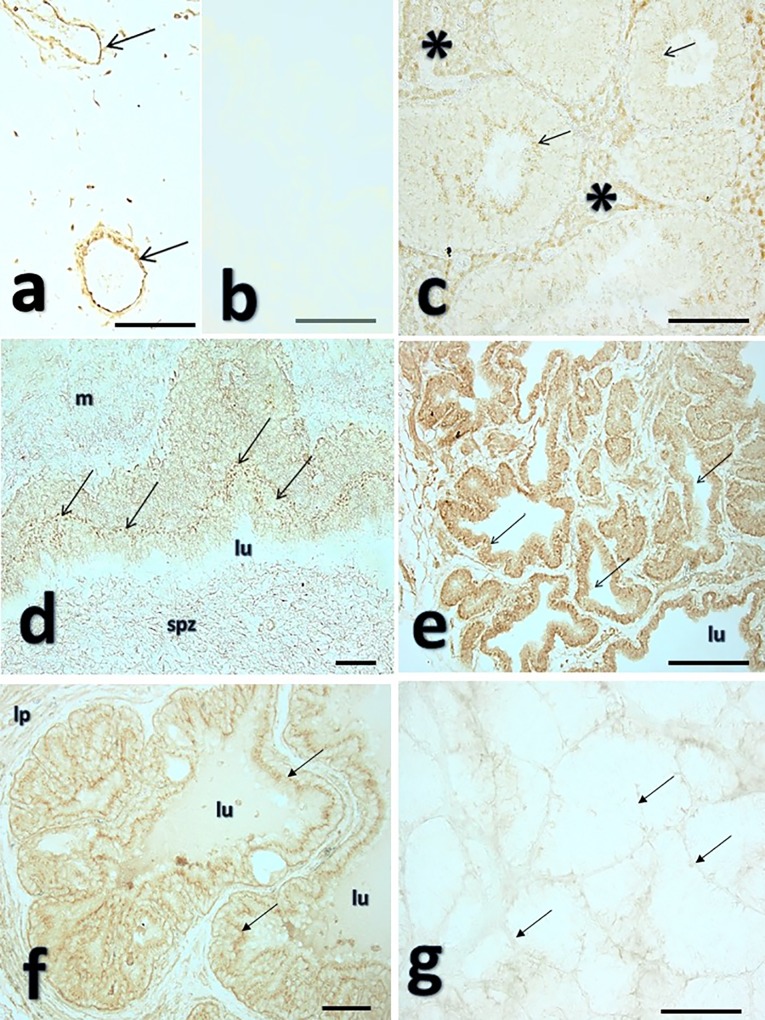
Immunolocalization of glutathione peroxidase-5 (GPX5) in boar genital organs. Fig 4a depicts a representative positive control section depicting immunostained blood vessels with a major restricted endothelial GPX-5 localization (arrows). A representative negative control prostate section (primary Ab omitted) is depicted in Fig 4b. Fig 4c shows a representative section of boar testis, showing immunostaining in the interstitium (blood vessels and Leydig cells, asterisk) and the seminiferous tubules, with a marked immunostaining in the elongated spermatids (arrows). The immunostaining was more marked in the lining epithelium of the ductus epididymides (Fig 4d, cauda segment) where both the principal epithelial cells (arrows point to the apical cell region, base of the stereocilia) and the luminal spermatozoa (spz) appeared stained, while the surrounding smooth muscle appears only faibly stained (m), lu: lumen. Fig 4e-g depict accessory sexual glands secretory epithelia of the prostate (4e), the seminal vesicles (4f) and the bulbourethral gland (4g). While prostate depicted both nuclear and cytoplasmic staining (4e), the seminal vesicles depicted mainly cytoplasmic staining (arrows) and a clear staining in the luminal secretion (lu), lp: lamina propria. The bulbourethral gland (4g) was immunostained in the blood vessel dominated interstitial with some staining in the latero-basal epithelial membrane (arrows). Not-counterstained sections, viewed with phase-contrast optics. Bars: 50μm.

The WB confirmed the presence of GPX-5 in boar testis, epididymis, cauda epididymis, seminal vesicles, prostate and bulbourethral glands extracted proteins as two bands of 64 and 51 KDa (Fig 3). The GPX-5 protein was hereby also immunolocalized using a polyclonal antibody in the genital organs of the male pig, including the spermatozoa present in the epididymal lumen. The positive control (oral cavity, blood vessels in the lamina propria) appeared, as expected, immunostained (Fig 4A). Negative controls (omission of the primary antibody) appeared unstained (see representative section in Fig 4B). The boar testis showed consistently marked staining of the interstitium, predominantly on the blood vessels and the Leydig cells. The seminiferous tubules presented a weak, homogeneous immunostaining of the epithelium, with scattered labelling among various stages of the germ cells, but with a more intense staining in the Sertoli cell cytoplasm and the elongated spermatids (Fig 4C). As expected, the GPX-5 was clearly localized in the epithelium lining the ductus epididymis, mainly on the principal cells. The immunostaining was mostly intracytoplasmic (including the stereocilia), but both the basal and particularly the apical membranes were markedly stained. The spermatozoa in the lumen were also stained (Fig 4D). Among the accessory sexual glands, the prostate and the seminal vesicles displayed strong immunostaining of the glandular and duct epithelia. The prostate epithelium depicted a clear nuclear and cytoplasmatic localization (Fig 4E), while the seminal vesicle immunostaining was mainly cytoplasmic (Fig 4F). Noteworthy, only the seminal vesicle secretion was immunostained (Fig 4F). The immunolocalization pattern changed in the bulbourethral gland (Fig 4G) where the immunolocalization in the glandular epithelium was conspicuously membrane-related with staining restricted to the basal and lateral epithelial membranes. In all sections, expectedly, vascular endothelia were markedly stained. There were no obvious differences in staining distribution or subjective intensity (++) among boars.

### Experiment 3: Relationship between SP-GPX5 concentration and sperm quality of liquid-stored semen samples

**Fig 5:**
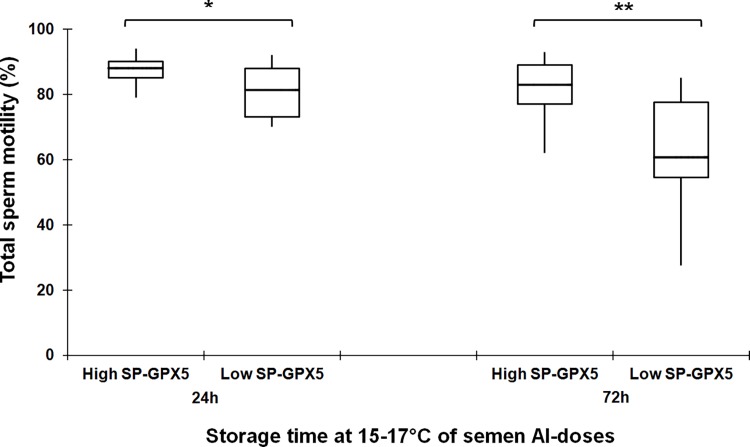
Relationship between the boar seminal plasma concentration of Glutathione Peroxidase 5 (SP-GPX5) and total sperm motility. Box-whisker plot showing total sperm motility of artificial insemination semen doses stored for 24 and 72 h at 15–17°C from boar ejaculates hierarchically grouped according to the concentration of SP-GPX5 as high (from 20.74 to 30.14 ng/mL, 15 ejaculates) and low (from 9.64 to 20.04 ng/mL, 29 ejaculates). (*) and (**) indicate significant differences (P < 0.01) and (P < 0.001), respectively, among different SP-GPX5 groups in each storage time.

The 44 extended semen samples were stored at 15–17°C and sperm quality was evaluated at 24 and 72 h of storage. The SP-GPX5 concentration differed among ejaculates, ranging from 9.63 ng/mL to 30.13 ng/mL. The ejaculates were classified (hierarchical clustering, P < 0.001) into 2 groups as with high (from 20.74 to 30.14 ng/mL, n = 15) or low (from 9.64 to 20.04 ng/mL, n = 29) SP-GPX5 concentrations. The sperm quality, in terms of total sperm motility, progressive sperm motility and sperm viability, decreased (P < 0.001) across storage time, irrespective of SP-GPX5 group. The total motility differed among SP-GPX5 groups at 24 h (P < 0.01) and at 72 h of storage (P < 0.001), showing the semen samples from ejaculates with high SP-GPX5 concentrations the highest percentage of total motile sperm (Fig 5). Progressive sperm motility and sperm viability did not differ between semen samples of the two SP-GPX5 groups, neither at 24 nor at 72 h of storage. The percentages of progressive sperm in the high and low SP-GPX5 groups were, respectively, 39.73 ± 2.52 and 40.86 ± 1.71 at 24 h, and 43.53 ± 3.01 and 37.34 ± 2.82 at 72 h of storage at 15–17°C. The percentages of viable sperm were 92.63 ± 0.93 and 90.98 ± 1.09 in the high SP-GPX5 group, and 91.06 ± 0.77 and 89.63 ± 0.79 in the low SP-GPX5 group, at 24 and 72 h of storage at 15–17°C, respectively.

### Experiment 4: Relationship between SP-GPX5 concentration and fertility post-AI of liquid-stored semen samples

**Fig 6:**
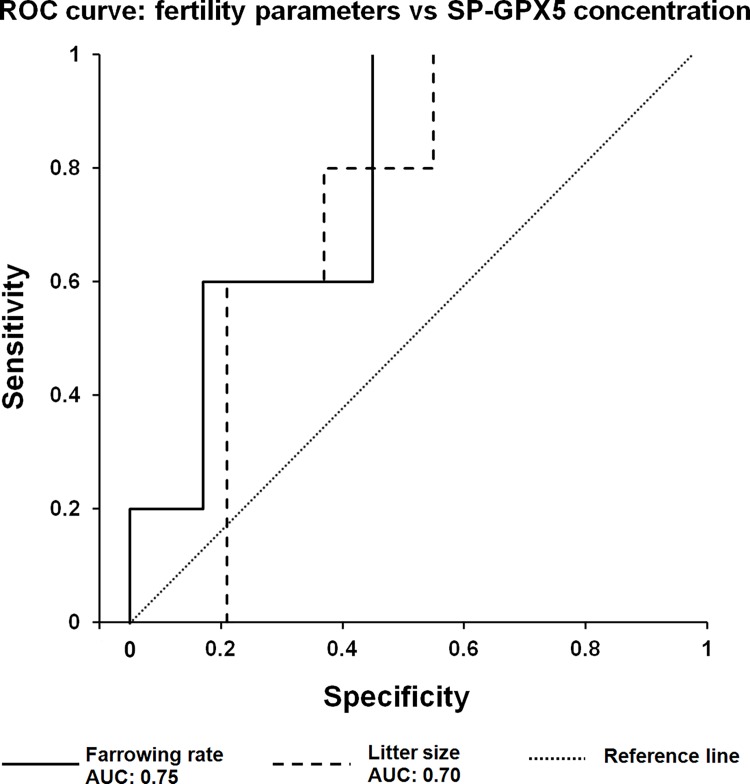
Potential boar fertility biomarker of boar seminal plasma Glutathione Peroxidase 5 (SP-GPX5). Nonparametric Receivers Operating Characteristic (ROC) curves showing the ability of boar SP-GPX5 concentration to predict farrowing rates and litter size of artificial insemination semen doses stored 24–72 h at 15–17°C. AUC: area under the ROC curve.

The SP-GPX5 concentration and fertility outcomes of AI-doses are summarized in Table 1. The boar population was classified (hierarchical clustering, P < 0.001) into two groups as having high or low SP-GPX5 concentrations. The SP-GPX5 concentration influenced fertility outcomes, showing overall highest farrowing rates (P < 0.01) and litter sizes (P < 0.01) the boars with high SP-GPX5 concentrations. However, regarding the ability of the SP-GPX5 concentrations to predict whether a boar will exhibit high fertility outcomes, the ROC curve (Fig 6) revealed that the SP-GPX5 concentrations had a fair discriminatory value for both farrowing rate (AUC = 0.75, P < 0.01) and litter size (AUC = 0.70, P < 0.05).

**Table 1:** Glutathione peroxidase 5 concentration in seminal plasma (SP-GPX5, ng/mL) and fertility AI-outcomes of the liquid stored semen doses (2,500 x 10^6^ sperm/dose). The semen doses from to entire ejaculates of eleven boars hierarchically grouped as high (in grey) and low (in white) SP-GPX5 concentration (4 entire ejaculates per boar).

Boars	Mean SP-GPX5 ± SEM (range)	Sows inseminated (n°)	Farrowing Rate (%)	Total Piglets Born (mean ± SEM)
a	18.02 ± 1.57 (14.75–20.87)	586	88.33 ± 1.70	13.06 ± 0.04
b	17.19 ± 1.22 (14.24–20.17)	605	89.63 ± 1.47	13.67 ± 0.04
c	15.61 ± 0.57 (14.20–16.76)	556	85.43 ± 1.50	13.55 ± 0.05
d	13.58 ± 1.30 (10.91–16.45)	334	88.44 ± 2.25	13.71 ± 0.06
e	13.14 ± 1.95 (8.68–17.86)	397	85.60 ± 1.99	13.79 ± 0.05
f	10.26 ± 0.26 (9.5–10.64)	567	82.49 ± 1.88	12.27 ± 0.05
g	10.16 ± 0.42 (9.40–11.77)	174	84.48 ± 2.75	13.82 ± 0.08
h	10.04 ± 0.54 (8.96–10.97)	258	79.84 ± 2.50	13.10 ± 0.08
i	9.38 ± 0.61 (8.69–10.60)	215	87.95 ± 3.00	12.14 ± 0.08
j	8.17 ± 0.57 (6.85–9.51)	904	86.75 ± 1.29	13.63 ± 0.04
k	6.62 ± 0.33 (6.27–7.61)	679	88.79 ± 1.57	12.45 ± 0.05

## Discussion

The data reported in the present study clearly demonstrated differences in SP-GPX5 concentration among boars, ejaculates within boars and different portions of boar ejaculate. The WB and the IHC analyses demonstrated, for the first time, that all organs of the boar genital tract expressed GPX5. Finally, this study also evidenced that SP-GPX5 concentration was positively related with total sperm motility and fertility outcomes in terms of farrowing rate and litter size.

The first experiment of this study showed that SP-GPX5 varies significantly among boars and among ejaculates within each boar. These results were expected, because previously Vilagran et al. [17] found quantitative differences in SP-GPX5 concentration among boar ejaculates with good and bad sperm freezability. In addition, the present results would also be in agreement with those achieved in previous studies in our laboratory that demonstrated inter- and intra-boar variability in the SP-TAC [6] and also of a particular SP-antioxidant enzyme: paraoxonase 1 [18]. Accordingly, it has been demonstrated that the expression of GPX5 in epididymis can vary in relation to several epididymal and testicular factors, fibroblast growth factor among other factors, and it also depends of androgen levels [11]. Differences in SP-GPX5 concentration were also evidenced among ejaculate portions, showing the first 10 mL of SRF the lowest concentration, without differences seen between the SRF and the post-SRF. Similarly, other SP-components also vary among boar ejaculate portions, for instance, the above mentioned TAC and paraoxonase 1 [6,18]. Different origins of the SP collected in each ejaculate fraction could explain these differences among ejaculate fractions [12]. However, it is noteworthy that the lowest SP-GPX5 concentration was found in the first 10 mL of SRF, whose SP comes mainly from the epididymis [19] particularly considering the epididymis was so far the only known site of origin for SP-GPX5 [11]. In order to clarify this apparent contradiction, a second experiment, using WB and IHC of different organs of the boar genital tract, was issued searching for the specific sites of GPX5 expression. The localization of GPX5 in the male genital tract has been previously described in mice [20], rats [21] and bulls [22], being the epididymis the sole male genital organ expressing GPX5. Specifically, GPX5 has been found in the principal epididymal cells lining, free in the epididymal lumen, linked to epididymosomes or bound to sperm transiting the epididymis, particularly located to the sub-acrosome membrane region [20, 23–25]. Accordingly, if the epididymis was the exclusive site of GPX5 expression also in pigs, the highest SP-GPX5 concentration should be found in the first 10 mL of SRF, which was definitively not the case. The WB and IHC revealed for the first time in a mammalian species, using a specific polyclonal antibody and performed for all organs of the genital tract of healthy, sexually mature and fertile boars, that GPX5 is expressed in other organs of the male genital tract in addition to the epididymis, including testis and all accessory sexual glands. In this regard, a former study demonstrated the presence of one unspecified GPX, in a band of 20kDa, in epithelial cells, vacuole membranes and vascular endothelium of boar seminal vesicle, prostate and bulbourethral glands [26]. The present study confirms such findings, demonstrating that the unspecific GPX would be GPX5, which was clearly expressed in boar accessory sexual glands; specifically the localization was nuclear and cytoplasmic in the prostate, mainly cytoplasmic in the seminal vesicle and membrane-related in the bulbourethral glands. This finding would provide support to the results achieved in the first experiment, which demonstrated that the concentration of SP-GPX5 was higher in rest of SRF and post-SRF (ejaculate portions with SP essentially from accessory sexual glands) than the first 10 mL of SRF (SP essentially derived from the cauda epididymis). One of the main findings of this study was the expression of GPX5 in boar testicular tissue, particularly in the Leydig cells and interstitium. Until now, GPX4 had been the unique GPX identified in testes, specifically in humans [27]. The expression of GPX5 in boar testicular tissues could indicate its contribution in the functional development of spermatozoa, probably involved in the protection of developing sperm against OS.

The third experiment focused in the relation between SP-GPX5 and sperm quality of liquid semen AI-doses stored at 15–17°C during 72h, storage time commonly used in commercial swine AI programs. The results showed a positive relationship between SP-GPX5 and the total number of motile sperm. Recently, in a review report about the effect of dead sperm on contemporary viable sperm, Roca et al. [28] highlighted that the damaging pathway followed by extracellular H_2_O_2_ present in boar semen samples and generated mainly from dead sperm, included sequentially LPO and motility loss in viable sperm. Unfortunately, the extracellular H_2_O_2_ in the liquid-stored semen AI-doses was not measured in the present study, but the subtle but statistically significant differences in the concentration of SP-GPX5 among semen AI-doses could reveal differences in the ability of SP to mitigate the harmful effects of extracellular H_2_O_2_, which would be reflected in differences in sperm motility rates, particularly in semen AI-doses stored for longer (72 h). Recently, Vilagran et al. [13] reported a negative correlation between SP-GPX5 and sperm quality, specifically motility and sperm membrane integrity, a surprising finding considering the known functions of SP-GPX5 on sperm quality and functionality [29]. The results of the present study disagree with this report, probably in relation to differences in the evaluation time which, in the Vilagran study, was done immediately after ejaculation. Our results indicate that the positive effectiveness of SP-GPX5 on sperm quality is particularly evidenced when liquid semen is stored, during 72h. Moreover, Vilagran et al. [13] identified SP-GPX5 using Western blotting but not by immunohistochemistry, as done in the present study to determine both presence and further, tissue localization.

The last experiment carried out in the present study, focusing on finding possible relationships between the SP-GPX5 concentration and AI-fertility outcomes, evidenced a positive relationship of SP-GPX5 concentration of liquid-stored semen AI-doses with farrowing rates and litter sizes of AI-sows. Novak et al. [12] demonstrated a positive relationship between SP-GPX5 and farrowing rates in pigs, although the number of boars and inseminated gilts/boar was minimal (4 respectively 50/boar). The present study, with 11 AI-boars evaluated and with more than 5,000 sows inseminated, ought to be more solid, confirmed a positive relationship between SP-GPX5 and farrowing rates. Moreover, the present results also demonstrated a positive relationship between SP-GPX5 concentration and litter size. Together, the results highlight a positive effect of SP-GPX5 on the capacity of sperm included in liquid-stored boar semen AI-doses to fertilize and initiate embryo development. These results are expected, as SP-GPX5 is positively related with the sperm quality of liquid-stored AI-doses, particularly with sperm motility, where the highest sperm motility rates resulted in highest *in vivo* fertility [30]. However, the way by which SP-GPX5 promotes *in vivo* fertility of boar sperm could go beyond a direct effect on sperm motility. In mice, a GPX5-KO model demonstrated that the lack of GPX5 was associated with infertility, specifically with embryo-fetal defects, miscarriages and perinatal mortality [31]. Immunofluorescence analysis of rat sperm demonstrated that SP-GPX5 was able to bind to the sperm head during epididymal transit and ejaculation [21], hypothesizing that this enzyme could prevent premature acrosome reactions once sperm are delivered into the female reproductive tract by binding GPX5 to membrane lipid peroxides, preventing the interaction between these peroxides and phospholipase A2 [32]. Moreover, spermatozoa that have never been in contact with GPX5 are more sensitive to nuclear DNA damage and, consequently, unable to develop healthy embryos, as demonstrated in GPX5-/- male mice [31], which could indicate that SP-GPX5 would have a positive impact on litter size in AI-sows [33], as it seems to be the case in the present study. In addition, SP-GPX5 appears free in the uterine lumen of mice after mating [20]. Consequently, SP-GPX5 seems to protect boar sperm even after their entry into the female genital tract, by potentially mitigating the putative damaging effects of ROS generated by uterine tissues and, thereby, by facilitating oocyte fertilization [34].

In summary, the present study demonstrates that all organs of the boar genital tract, including testis, epididymis and accessory sexual glands, are able to express GPX5, which is present in seminal plasma in variable concentration depending on boar, ejaculate and even ejaculate portions within boar. The present study also evidences a positive relationship between SP-GPX5 and both sperm quality, particularly total sperm motility, and *in vivo* fertility outcomes of liquid-stored semen AI-doses. These relationships would suggest that the concentration of SP-GPX5 could be considered as potential sperm quality and fertility biomarker for AI-boars. However, before the last suggestion can be considered, more AI-trials using larger cohorts of tested boars and inseminated sows should be carried out, as the ROC curves in the present study, where *in vivo* fertility of 11 boars was evaluated, just demonstrated a fair discriminatory value of SP-GPX5 to both farrowing rate and litter size. Anyway, the present results suggest GPX5 to be considered as a semen extender additive to improve sperm quality and fertility of liquid-stored boar semen.

## References

[1] Radomil L, Pettitt MJ, Merkies KM, Hickey KD, Buhr MM. Stress and dietary factors modify boar sperm for processing. Reprod Domest Anim. 2011;46: 39–44. 10.1111/j.1439-0531.2011.01865.x 21884275

[2] Tvrdá E, Kňažická Z, Bárdos L, Massányi P, Lukáč N. Impact of oxidative stress on male fertility—a review. Acta Vet Hung. 2011;59: 465–84. 10.1556/AVet.2011.034 22079708

[3] Rodriguez-Martinez H, Saravia F, Wallgren M, Roca J, Peña FJ. Influence of seminal plasma on the kinematics of boar spermatozoa during freezing. Theriogenology. 2008;70: 1242–1250. 10.1016/j.theriogenology.2008.06.007 18639331

[4] Juyena NS, Stelletta C. Seminal plasma: an essential attribute to spermatozoa. J Androl. 2012;33: 536–551. 10.2164/jandrol.110.012583 22016346

[5] Fraga CG, Oteiza PI, Galleano M. In vitro measurements and interpretation of total antioxidant capacity. Biochim Biophys Acta. 2014;1840: 931–934. 10.1016/j.bbagen.2013.06.030 23830861

[6] Barranco I, Tvarijonaviciute A, Perez-Patiño C, Parrilla I, Ceron JJ, Martinez EA et al High total antioxidant capacity of the porcine seminal plasma (SP-TAC) relates to sperm survival and fertility. Sci Rep. 2015;5: 18538 10.1038/srep18538 26688188 PMC4685244

[7] Guthrie HD, Welch GR. Determination of intracellular reactive oxygen species and high mitochondrial membrane potential in Percoll-treated viable boar sperm using fluorescence-activated flow cytometry. J Anim Sci. 2006;84: 2089–2100. 10.2527/jas.2005-766 16864869

[8] Awda BJ, Mackenzie-Bell M, Buhr MM. Reactive oxygen species and boar sperm function. Biol Reprod. 2009;81: 553–561. 10.1095/biolreprod.109.076471 19357363

[9] Chabory E, Damon C, Lenoir A, Henry-Berger J, Vernet P, Cadet R et al Mammalian glutathione peroxidases control acquisition and maintenance of spermatozoa integrity. J Anim Sci. 2010;88: 1321–1331. 10.2527/jas.2009-2583 20042549

[10] Noblanc A, Kocer A, Chabory E, Vernet P, Saez F, Cadet R et al Glutathione peroxidases at work on epididymal spermatozoa: an example of the dual effect of reactive oxygen species on mammalian male fertilizing ability. J Androl. 2011;32: 641–650. 10.2164/jandrol.110.012823 21441427

[11] Brigelius-Flohé R, Maiorino M. Glutathione peroxidases. Biochim Biophys Acta. 2013;1830: 3289–3303. 10.1016/j.bbagen.2012.11.020 23201771

[12] Novak S, Ruiz-Sánchez A, Dixon WT, Foxcroft GR, Dyck MK. Seminal plasma proteins as potential markers of relative fertility in boars. J Androl. 2010;31: 188–200. 10.2164/jandrol.109.007583 19713565

[13] Vilagran I, Castillo-Martín M, Prieto-Martínez N, Bonet S, Yeste M. Triosephosphate isomerase (TPI) and epididymal secretory glutathione peroxidase (GPX5) are markers for boar sperm quality. Anim Reprod Sci. 2016;165: 22–30. 10.1016/j.anireprosci.2015.12.001 26711247

[14] Roca J, Parrilla I, Bolarin A, Martinez EA, Rodriguez-Martinez H. Will AI in pigs become more efficient? Theriogenology. 2015; pii: S0093-691X(15)00651-2. 10.1016/j.theriogenology.2015.11.026 26723133

[15] Vicente-Carrillo A, Alvarez-Rodriguez M, Rodriguez-Martinez H. The mu (μ) and delta (δ) opioid receptors modulate boar sperm motility. Mol Reprod Dev. 2016 (in press). 10.1002/mrd.22675 27391529

[16] Broekhuijse ML, Šoštarić E, Feitsma H, Gadella BM. Relationship of flow cytometric sperm integrity assessments with boar fertility performance under optimized field conditions. J Anim Sci. 2012;90: 4327–4336. 10.2527/jas.2012-5040 23255815

[17] Vilagran I, Yeste M, Sancho S, Castillo J, Oliva R, Bonet S. Comparative analysis of boar seminal plasma proteome from different freezability ejaculates and identification of Fibronectin 1 as sperm freezability marker. Andrology. 2015;3: 345–356. 10.1111/andr.12009 25678437

[18] Barranco I, Tvarijonaviciute A, Perez-Patiño C, Alkmin DV, Ceron JJ, Martinez EA et al The activity of paraoxonase type 1 (PON-1) in boar seminal plasma and its relationship with sperm quality, functionality, and in vivo fertility. Andrology. 2015;3: 315–320. 10.1111/andr.309 25598515

[19] Rodriguez-Martinez H, Kvist U, Ernerudh J, Sanz L, Calvete JJ. Seminal plasma proteins: what role do they play? Am J Reprod Immunol. 2011;66: 11–22. 10.1111/j.1600-0897.2011.01033.x 21726334

[20] Vernet P, Faure J, Dufaure JP, Drevet JR. Tissue and developmental distribution, dependence upon testicular factors and attachment to spermatozoa of GPX5, a murine epididymis-specific glutathione peroxidase. Mol Reprod Dev. 1997;47: 87–98. 10.1002/(SICI)1098-2795(199705)47:1<87::AID-MRD12>3.0.CO;2-X 9110319

[21] Williams K, Frayne J, Hall L. Expression of extracellular glutathione peroxidase type 5 (GPX5) in the rat male reproductive tract. Mol Hum Reprod. 1998;4: 841–848. 10.1093/molehr/4.9.841 9783843

[22] Grignard E, Morin J, Vernet P, Drevet JR. GPX5 orthologs of the mouse epididymis-restricted and sperm-bound selenium-independent glutathione peroxidase are not expressed with the same quantitative and spatial characteristics in large domestic animals. Theriogenology. 2005;64: 1016–1033. 10.1016/j.theriogenology.2005.01.008 16054503

[23] Rejraji H, Vernet P, Drevet JR. GPX5 is present in the mouse caput and cauda epididymidis lumen at three different locations. Mol Reprod Dev. 2002;63: 96–103. 10.1002/mrd.10136 12211066

[24] Drevet JR. The antioxidant glutathione peroxidase family and spermatozoa: a complex story. Mol Cell Endocrinol. 2006;250: 70–79. 10.1016/j.mce.2005.12.027 16427183

[25] Zhang T, Chabory E, Britan A, Grignard E, Pitiot O et al GPX5, the selenium-independent glutathione peroxidase-encoding single copy gene is differentially expressed in mouse epididymis. Reprod Fertil Dev. 2008;20: 615–625. 10.1071/RD08008 18577359

[26] Jelezarsky L, Vaisberg Ch, Chaushev T, Sapundjiev E. Localization and characterization of glutathione peroxidase (GPx) in boar accessory sex glands, seminal plasma, and spermatozoa and activity of GPx in boar semen. Theriogenology. 2008;69: 139–145. 10.1016/j.theriogenology.2007.08.016 17964641

[27] Moreno SG, Laux G, Brielmeier M, Bornkamm GW, Conrad M. Testis-specific expression of the nuclear form of phospholipid hydroperoxide glutathione peroxidase (PHGPx). Biol Chem. 2003;384: 635–643. 10.1515/BC.2003.070 12751792

[28] Roca J, Parrilla I, Gil MA, Cuello C, Martinez EA et al Non-viable sperm in the ejaculate: Lethal escorts for contemporary viable sperm. Anim Reprod Sci. 2016 10.1016/j.anireprosci.2016.02.028 26948922

[29] Gharagozloo P, Gutiérrez-Adán A, Champroux A, Noblanc A, Kocer A, Calle A et al A novel antioxidant formulation designed to treat male infertility associated with oxidative stress: promising preclinical evidence from animal models. Hum Reprod. 2016;31: 252–262. 10.1093/humrep/dev302 26732620

[30] Popwell JM, Flowers WL. Variability in relationships between semen quality and estimates of in vivo and in vitro fertility in boars. Anim Reprod Sci. 2004;81: 97–113. 10.1016/j.anireprosci.2003.08.007 14749052

[31] Chabory E, Damon C, Lenoir A, Kauselmann G, Kern H, Zevnik B et al Epididymis seleno-independent glutathione peroxidase 5 maintains sperm DNA integrity in mice. J Clin Invest. 2009;119: 2074–2085. 10.1172/JCI38940 19546506 PMC2701883

[32] Okamura N, Iwaki Y, Hiramoto S, Tamba M, Bannai S, Sugita Y et al Molecular cloning and characterization of the epididymis-specific glutathione peroxidase-like protein secreted in the porcine epididymal fluid. Biochim Biophys Acta. 1997;1336: 99–109. 10.1016/S0304-4165(97)00016-0 9271255

[33] Boe-Hansen GB, Christensen P, Vibjerg D, Nielsen MB, Hedeboe AM. Sperm chromatin structure integrity in liquid stored boar semen and its relationships with field fertility. Theriogenology. 2008;69: 728–736. 10.1016/j.theriogenology.2007.12.004 18242673

[34] Agarwal A, Aponte-Mellado A, Premkumar BJ, Shaman A, Gupta S. The effects of oxidative stress on female reproduction: a review. Reprod Biol Endocrinol. 2012;10: 49 10.1186/1477-7827-10-49 22748101 PMC3527168

